# Genetic architecture and molecular regulation of sorghum domestication

**DOI:** 10.1007/s42994-022-00089-y

**Published:** 2022-12-19

**Authors:** Fengyong Ge, Peng Xie, Yaorong Wu, Qi Xie

**Affiliations:** 1 State Key Laboratory of Plant Genomics, Institute of Genetics and Developmental Biology, The Innovative Academy of Seed Design, Chinese Academy of Sciences, Beijing, 100101 China; 2 University of Chinese Academy of Sciences, Beijing, 100049 China

**Keywords:** Domestication, Genetic basis, Molecular mechanism, Genes, Sorghum

## Abstract

Over time, wild crops have been domesticated by humans, and the knowledge gained from parallel selection and convergent domestication-related studies in cereals has contributed to current techniques used in molecular plant breeding. Sorghum (*Sorghum bicolor* (L.) Moench) is the world’s fifth-most popular cereal crop and was one of the first crops cultivated by ancient farmers. In recent years, genetic and genomic studies have provided a better understanding of sorghum domestication and improvements. Here, we discuss the origin, diversification, and domestication processes of sorghum based on archeological discoveries and genomic analyses. This review also comprehensively summarized the genetic basis of key genes related to sorghum domestication and outlined their molecular mechanisms. It highlights that the absence of a domestication bottleneck in sorghum is the result of both evolution and human selection. Additionally, understanding beneficial alleles and their molecular interactions will allow us to quickly design new varieties by further de novo domestication.

## Introduction

Crop domestication has contributed to the rise of agriculture and the transition away from hunting and gathering, laying the groundwork for modern human civilization (Purugganan and Fuller 2009). Evidence collected from archeological discoveries and genetic and genomic studies indicates that crop domestication is a protracted process rather than a short, discrete event (Purugganan 2019). In general, the domestication process can be divided into four stages: in stage 1, humans harvest and consume wild plants; in stage 2, humans deliberately cultivate crops, resulting in genetic bottlenecks and a decline in wild alleles (Doebley et al. 2006); in stage 3, domesticated plants expand to new geographical regions and adapt to the local environment, resulting in a diversification of domesticated alleles and the formation of landraces; in stage 4, humans deliberately breed improved cultivars to meet the demands of modern diets (Gaut et al. 2018; Lenser and Theissen 2013). Compared with their wild ancestors, domesticated crops share some common morphological and physiological characteristics, which is known as domestication syndrome, including loss of seed shattering (non-shattering), loss of seed dormancy, seed enlargement, synchronous germination, and changes in tiller number and stature (Gepts 2014; Stetter et al. 2017). These traits typically occur under parallel/convergent domestication among different crops (Lenser and Theissen 2013; Purugganan 2019). The plant domestication center refers to the original geographic region where a specific species was domesticated. Three main cereal crops (rice, wheat, and maize) supply more than 50% of human calories, out of ~ 5500 food crops cultivated worldwide (Ross-Ibarra et al. 2007; Zhao et al. 2021), and were originally domesticated in East Asia, the Middle East, and Central America, respectively (Gepts 2014; Larson et al. 2014).

Sorghum (*Sorghum bicolor* (L.) Moench) is the fifth-most popular cereal crop in the world and is a staple food for more than 500 million people in Africa and Asia (Xin et al. 2021; FAOSTAT, https://www.fao.org/faostat/en/#data). It is estimated that sorghum was first cultivated as a food source in the Sahelian belt of Africa and was originally domesticated in central eastern Sudan approximately 6000 to 4000 years ago (Winchell et al. 2017). In addition to being a valuable source of calories, sorghum can also be used as fiber, forage, and fuel (Hao et al. 2021; Silva et al. 2021; Xie and Xu 2019). Since sorghum was domesticated and evolved in arid and semiarid ecosystems, it exhibits strong resistance to many abiotic stresses, such as drought, high light, barrenness, salt, and alkalinity, making it an ideal resistant plant resource to meet the modern demands of crop breeding to ensure food security under climate change (Ma et al. 2020; Prasad et al. 2021; Varoquaux et al. 2019; Xie and Xu 2019; Yang et al. 2020). Apart from the archeological and fossil discoveries, recent evidence from genetic and genomic studies of sorghum provides clues about its domestication at the molecular level (Baye et al. 2022; Wu et al. 2022, 2019; Xie et al. 2022; Zhang et al. 2018; Zhou et al. 2021). With the help of modern advanced biotechnologies, such as synthetic biology and gene editing techniques, we can rapidly improve current cultivars or create new crops by de novo domestication (such as in tomato and rice) to address the crises caused by food deficiency once we understand the underlying genetic basis of domestication-related genes (Li et al. 2018; Yu et al. 2021).

In this review, we discuss widely accepted opinions about sorghum domestication and characteristics based on archeological records and genomic studies. We comprehensively and systematically summarized the genetic basis and molecular mechanisms of major sorghum domestication-related genes. The conclusions regarding the artificial selection of sorghum will accelerate sorghum breeding processes when combined with advanced biotechnologies.

## Sorghum origin, distribution, and classification

Although some issues are still under debate, it is widely accepted that sorghum originated from Africa in approximately 7500 BC, based on evidence gathered from archeological discoveries (Ananda et al. 2020). Some studies have reported the potential evolutionary history and dispersal route of domesticated sorghum (Burgarella et al. 2021; Fuller and Stevens 2018; Venkateswaran et al. 2019; Winchell et al. 2018). It has been proposed that sorghum was first domesticated in the eastern Sahelian zone in approximately 4000 BC and propagated to South Asia approximately 1000 years later (Winchell et al. 2017, 2018). Then, sorghum was introduced to China and domesticated into Chinese kaoliang (Zhang and Ping 2022).

**Fig. 1:**
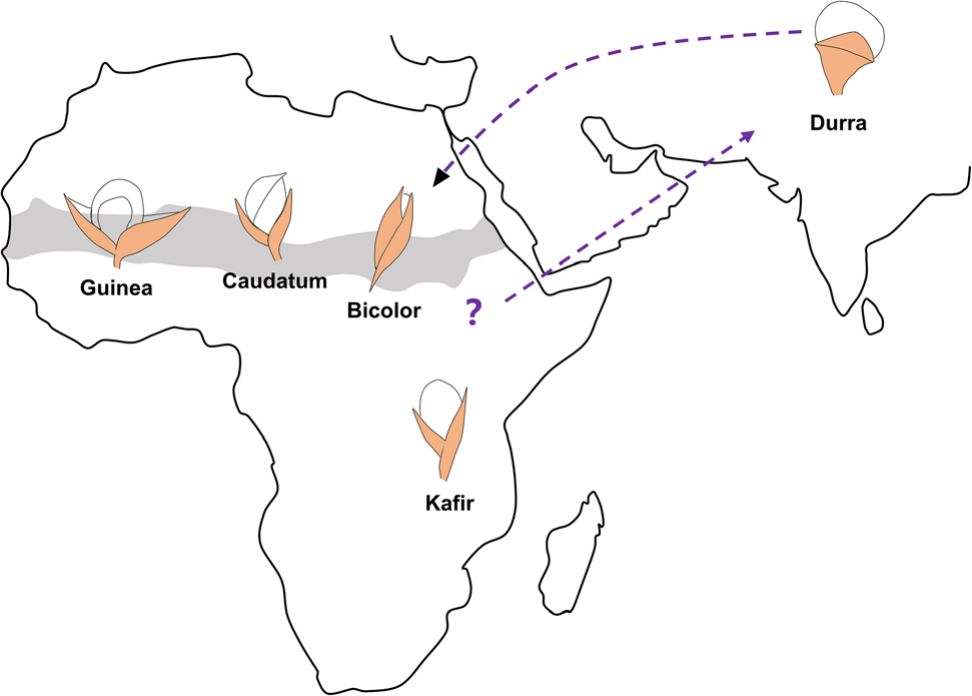
Domestication centers, original distribution, and spikelet morphology of five domesticated sorghum races (bicolor, guinea, caudatum, kafir, and durra). The early bicolor race was distributed in the eastern Sahelian zone and spread to other African regions. Its seeds are more tightly covered than those of the other four races. Race guinea probably evolved in West Africa and adapted to humid habitats with glumes opening at a large angle. Race kafir is widely grown in South Africa, with glumes varying in length. Race caudatum is dominant in eastern Nigeria, Sudan, and Uganda, with a relatively lower glume coverage. Race durra could be derived from the hybridization between race bicolor and the local wild species in India, and then subsequently re-introduced to Africa. Arrows indicate the possible spreading routes of race durra. The gray background represents the Sahelian zone

The genus *Sorghum* consists of 23 or 24 species, though the taxonomy of this genus is still being debated (Ananda et al. 2020; Ohadi et al. 2018). *Sorghum bicolor* (L.) Moench subsp. *bicolor* contains all cultivated sorghum varieties, which were derived from the wild progenitor *S. bicolor* subsp. *verticilliflorum* (formerly known as subsp. *arundinaceum*), which is widely distributed in Africa (Berenji et al. 2011; Wet and Harlan 1971; Wet and Huckabay 1967). Instead of the complex early classification that divided cultivated sorghum into 158 varieties, Harlan and de Wet proposed a revised version that has been accepted by the following researchers (Harlan and de Wet 1972). According to the simplified morphological characteristics of the spikelet and head type, cultivated sorghum can be classified into five basic races: bicolor, guinea, caudatum, kafir, and durra. Race bicolor is considered the oldest race, and its seeds are more tightly covered by glumes than those of the other four races (de Wet and Shechter 1977). Race guinea likely evolved in West Africa and adapted to humid habitats with a loose head type and glumes opening at a large angle (de Wet et al. 1972). Race durra could be derived from hybridization between the race bicolor and local wild species in India (Harlan and Stemler 1976). It has a compact panicle and lower glumes than bicolor. Glumes typically have different textures between the tip and the base (de Wet and Shechter 1977). Race kafir shows glumes varying in length, derived from early bicolor or independently evolved from the local wild race in South Africa. The origin of race caudatum has not been fully determined. It could be directly selected from early bicolor with lower glumes and has become a major sorghum donor widely distributed in eastern Nigeria, Sudan, and Uganda (Shechter and de Wet 1975). The proposed original domestication center, main distribution, and spikelet morphology of five cultivated sorghum races in Africa are depicted in Fig. 1.

During agricultural cultivation, sorghum can be classified into different subgroups based on its end use. In the literature, domesticated sorghum has been classified into grain sorghum, sweet sorghum, forage sorghum, and biomass sorghum (Silva et al. 2021). However, the boundary between forage and biomass sorghum is unclear, and another classification added broom sorghum instead of biomass sorghum (Li and Yu 2022). Grain sorghum typically has larger seeds and a short lifespan, while sweet sorghum accumulates abundant soluble sugars in its stem. Forage sorghum is bred to feed animals with higher biomass and better palatability. Broom sorghum with long panicles is used to produce traditional homemade brooms (Berenji et al. 2011). The various morphological characteristics of sorghum are due to evolution and artificial selection, indicating that there are abundant genetic resources related to these traits.

## Genomic footprints of sorghum domestication

Morphological variation during crop evolution is mainly determined by genetic modification in the nucleotide sequence of the genome. Sorghum is a diploid crop (2*n* = 2*x* = 20). The whole-genome assembly of the sorghum cultivar BTx623 was first achieved using de novo sequencing in 2009, and the reference genome size is approximately 730 Mb (Paterson et al. 2009). A subsequently improved genome assembly version annotated 34,211 genes (McCormick et al. 2018). Additionally, the reference genomes of the sorghum cultivar Tx430 and a sweet sorghum line Rio were also released in recent years (Cooper et al. 2019; Deschamps et al. 2018). The genome sequences of the other three sorghum accessions, BTx642, RTx430, and SC187, are also available on the Phytozome website (https://phytozome-next.jgi.doe.gov/). A comparison of genomic and transcriptomic data between BTx623 and Rio revealed structural variations and differentially expressed genes involved in sugar metabolism (Cooper et al. 2019). The pangenome of cultivated and wild sorghum revealed new annotated genes and presence and absence variations (PAVs) that could play an important role in sorghum domestication and diversification (Ruperao et al. 2021; Tao et al. 2021).

Advancements in sequencing technology and bioinformatics make it possible to analyze genetic information at the population level, increasing our knowledge of the evolutionary trajectory on many species, such as rice, soybean, tomato, lettuce, and apricots (Groppi et al. 2021; Lin et al. 2014; Lu et al. 2020; Qin et al. 2021; Wei et al. 2021). In sorghum, resequencing analysis of nine archeological accessions discovered at Qasr Ibrim from approximately 800 BC, as well as wild and cultivated sorghum genomes, revealed that there was no obvious domestication bottleneck in sorghum, but that there was a gradual decline in genetic diversity. It also induced an increase in deleterious mutations in cultivated sorghum lines, known as the “cost of domestication” (Gaut et al. 2018; Smith et al. 2019). Evidence from the genome information demonstrated the presence of occasional hybridization between the Asian durra type and African bicolor type, which led to genetic rescue and diversification in sorghum domestication. Hybridization or genetic introgression was also discovered between cultivated sorghum and their weedy or wild relatives, indicating the occurrence of high-frequency active gene flow in sorghum. This differs from other strict self-pollination crops (Ohadi et al. 2018).

A recent population genetic study deepened our understanding of sorghum domestication and diversification at the whole-genome level. A total of 445 sorghum accessions were classified into seven subpopulations, including wild, sudangrass, landrace broom (LB), landrace grain (LG), improved grain (IG), improved sweet (IS), and ambiguous lines (AL), based on distinct phenotypic differentiation. Analysis of whole-genome resequencing data also revealed the existence of frequent genetic exchanges between LG and the wild type. LB lines, mostly collected from Asia, received gene flow from the common ancestor with LG, while IS received gene flows from both LB and sudangrass (Wu et al. 2022). In addition, genomic regions with selection signals were identified in different groups, and eight models based on the haplotype changes of domesticated genes were proposed. Among them, two important models represented soft selection with multiple domestication origins and hard selection with only one domestication event. Deep whole-genome resequencing and analysis of a sorghum association panel (SAP) containing 400 sorghum accessions identified 18 genomic regions with significant *F*_*st*_ peaks. These peaks varied in the five typical domesticated races, which reflects evolutionary differences and relationships during sorghum domestication. More importantly, these regions overlap with some previously reported quantitative trait loci (QTLs), indicating that the potential genetic mechanism underlying domestication-related traits could be uncovered in the future (Boatwright et al. 2022).

## Genetic dissection of domestication-related traits in sorghum

**Fig. 2:**
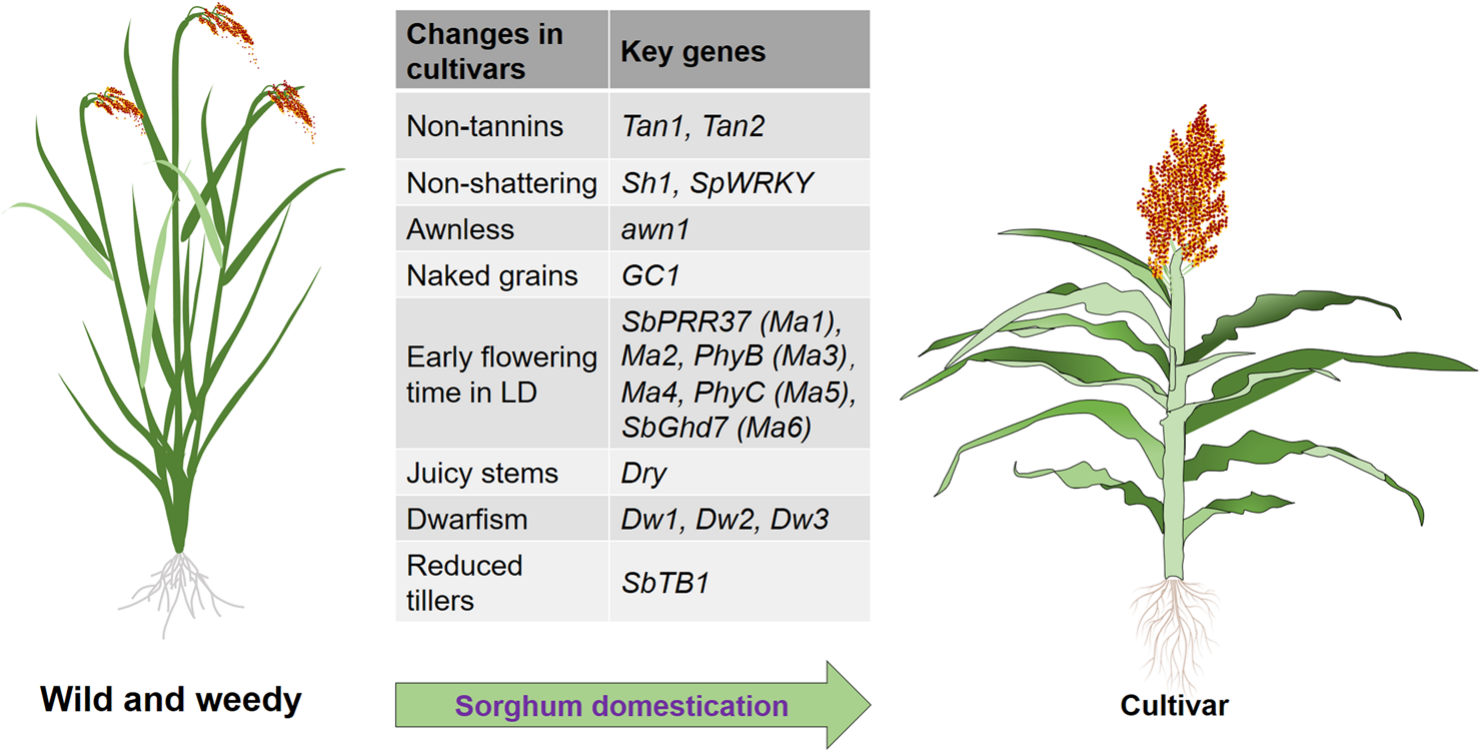
Plant architecture of wild and weedy sorghum and domesticated sorghum cultivar. The major genes controlling sorghum domestication-related traits are *SbTB1* (reduced tillers), *Dw1*, *Dw2,* and *Dw3* (dwarfism), *Dry* (stem juiciness), *SbPRR37*, *Ma2*, *PhyB*, *Ma4*, *PhyC*, and *SbGhd7* (early-flowering time in LD), *GC1* (naked grains), *awn1* (awnless), *Sh1* and *SpWRKY* (non-shattering), and *Tan1* and *Tan2* (non-tannin). *LD* long day, *Sb*, *Sorghum bicolor*

Studies analyzing the genetics and molecular biology of sorghum have identified many genes related to sorghum domestication, and the underlying genetic basis has been well elucidated. In the following text, we summarize the major domesticated genes in sorghum and introduce their molecular mechanisms. These genes are mostly involved in panicle-related traits, such as seed shattering, tannin content, awn and glume coverage, while others are related to stem juiciness, flowering, and plant architecture, including plant height and tiller number (Fig. 2 and Table 1).

**Table 1:** Genes that potentially underlie domestication, diversification and improvement of sorghum

Trait	Gene name	Gene loci	Gene category	Causative change	References
Plant height	*Dw1*	*Sobic.009G229800*	Uncharacterized	SNP in second exon causing premature stop	Yamaguchi et al. 2016
	*Dw2*	*Sobic.006G067700*	AGC protein kinase	2 bp deletion in first exon causing frameshift and truncated protein	Hilley et al. 2017; Oliver et al. 2021
	*Dw3*	*Sobic.007G163800*	ABC transporter	Duplication in fifth exon	Multani et al. 2003
	*Dw4*	Unknown	Unknown	NA	Quinby and Martin 1954
Tillers	*SbTB1*	*Sobic.001G121600*	TCP domain transcription factor	SNP in promoter	Kebrom et al. 2006; Wu et al. 2022
Stem juiciness	*Dry* or *D*	*Sobic.006G147400*	NAC domain transcription factor	Large deletion	Fujimoto et al. 2018; Zhang et al. 2018
Flowering	*SbPRR37* (*Ma1*)	*Sobic.006G057866*	PRR protein	*Sbprr37-1*: 1 bp deletion in first exon causing frameshift and truncated protein	Murphy et al. 2011
	*Ma2*	*Sobic.002G302700*	SMYD domain	SNP in third exon causing premature stop	Casto et al. 2019
	*PhyB* (*Ma3*)	*Sobic.001G394400*	Phytochrome B	*phyB-1* (*ma3*^*R*^): SNP in third exon causing premature stop	Childs et al. 1997; Yang et al. 2014a
	*Ma4*	unknown	Unknown	NA	Quinby et al. 1966
	*PhyC* (*Ma5*)	*Sobic.001G087100*	Phytochrome C	SNPs in exon causing nonsynonymous mutation	Yang et al. 2014a
	*SbGhd7* (*Ma6*)	Sobic.006G004400	CCT domain	*ghd7-1*: 5 bp insertion in first exon causing frameshift and truncated protein	Murphy et al. 2014
Awn	*Awn1*	*Sobic.003G421300*	ALOG domain	Translocation leading to a new promoter	Zhou et al. 2021
Glume coverage	*GC1*	*Sobic.001G341700*	Gγ subunit protein	*gc1-a*: 5 bp insertion in fifth exon causing frameshift and truncated protein	Xie et al. 2022
Tannin content	*Tan1*	*Sobic.004G280800*	WD40 domain	InDels in exon causing frameshift and truncated protein	Wu et al. 2019; Xie et al. 2019
	*Tan2*	*Sobic.002G076600*	bHLH transcription factor		
Shattering	*Sh1*	*Sobic.001G152901*	YABBY domain transcription factor	*SC265-like*: splicing variant *Tx430-like*: SNPs in promoter and intron *Tx623-like*: 2.2-kb deletion	Lin et al. 2012

Gene loci are based on *sorghum bicolor* v3.1.

*SNP* single nucleotide polymorphism, *InDel* insertion and deletion, *NA* information not available

### Plant height and tillers

Plant height is one of the most prominent target characteristics in crop breeding. Dwarfism in crops can increase lodging resistance and decrease grain yield loss. In rice and wheat, semidwarf varieties with deficiencies in gibberellin biosynthesis and signaling pathways were bred by breeders and subsequently initiated the “green revolution” (Wang et al. 2021). However, dwarfism is not a universal breeding goal for sweet or forage sorghum due to the demand for varieties with tall and robust statures and high biomass. Additionally, decreased plant height is widely selected in grain sorghum or other races with high grain yield.

It has been reported that sorghum plant height is controlled by four independent loci, named *Dw1*, *Dw2*, *Dw3*, and *Dw4* (Quinby and Martin 1954). Recessive mutations of each locus could lead to decreases in plant height. The underlying genes of the *Dw1*, *Dw2*, and *Dw3* loci have been cloned. *Dw3* encodes a protein containing transmembrane domains and ATP-binding domains. It functions in transporting auxin from the middle to lower stem tissues in a light-dependent manner. A nonfunctional allele with an 882 bp duplication in the fifth exon of *Dw3* was found in the *dw3* mutant. This mutation caused the dwarf phenotype with a reduction in internode length. However, the unequal crossing over of this duplication led to an unstable dwarf phenotype as a few accidental offspring individuals with tall plant height (Multani et al. 2003). *Dw1* was finely mapped to a 33 kb region on chromosome 6, and *Sobic.009G229800* was considered the candidate gene (Hilley et al. 2016). *Dw1* encodes a putative membrane protein with a higher expression level in elongating middle stems. The dwarf parent 80M carries the *dw1* allele with a truncated protein caused by an A to T mutation in the second exon (Yamaguchi et al. 2016). However, the molecular mechanism of *Dw1* remains unknown. The *Dw2* locus has been detected in many studies. Through fine-mapping from two recombinant inbred lines (RIL) populations, *Sobic.006G067700* was finally confirmed as the candidate gene instead of the previously reported gene *Sobic.006G067600* (Hilley et al. 2017). *Dw2* encodes a protein kinase belonging to the AGCVIII subfamily. The recessive *dw2* allele carries an Indel in the first exon, which causes a frameshift and a truncated protein. A subsequent study revealed that cell proliferation was repressed in NIL-DDYM (*dw2* allele), resulting in a shortened internode. In addition, mutation of *Dw2* caused irregular cell shapes and altered the morphology of vascular bundles. Cell wall polysaccharide components were also changed in *dw2*. Phosphoproteomic analysis demonstrated that *Dw2* was also involved in regulating lipid signaling and endomembrane trafficking (Oliver et al. 2021). Genome-wide selection signal detection found that the *Dw2* region has a much higher *F*_*st*_ value in the improved accessions than in the five basic races, indicating that *Dw2* was under positive selection in crop improvement practice after domestication (Boatwright et al. 2022). Other plant height-related regions were also detected in a genome-wide association study (GWAS), but it is unclear whether the genes are related to domestication.

Another agronomic trait related to shoot architecture is tiller number. Compared with their wild ancestors, domesticated and improved crop cultivars exhibit a great reduction in tiller number due to the benefits of close planting. *TB1*, a basic helix-loop-helix transcription factor negatively regulating tiller number, was first cloned in maize, and its orthologous gene in rice or sorghum plays similar roles (Doebley et al. 1997). A higher expression level of *SbTB1* observed in the sorghum *phyB-1* mutant revealed the relationship between tiller development and light signals (Kebrom et al. 2006). A population genetic study proved that the *SbTB1* region was under strong selection during sorghum domestication. All sorghum cultivars carry the same haplotype, while variations in the promoter region of *SbTB1* could explain the changes in tiller number between wild and domesticated species (Wu et al. 2022).

### Stem juiciness

The crop stem parenchyma cells play a role in storing water and nutrients, but become dry or form cavities surrounded by epidermal cells at the mature stage. Grain sorghum and sweet sorghum exhibit distinct stem morphology: grain sorghum stems become dry and pithy, while sweet sorghum stems maintain much higher water content and are juicy. This absorbing phenotype in sorghum has been investigated since last century (Swanson and Parker 1931). Early studies suggested that pithy or juicy stems in sorghum were determined by a single *D* locus, and that pithy was dominant to juicy. It has also been observed that pithy and juicy stems were closely associated with white and green midribs, respectively. In 2001, Hart et al. reported that the *D* locus was cosegregated with the *Xtxp97* marker in a sorghum genetic map (Hart et al. 2001). Both later GWAS and bulked segregant analysis (BSA) have mapped the *D* locus, and another study narrowed the *D* locus region with only six genes inside by map-based cloning (Han et al. 2015; Upadhyaya et al. 2022; Zhai et al. 2014). However, the underlying gene and its role in the origin of sweet sorghum remained obscure until two 2018 studies.

Using an F_2_ population derived from a cross between a dry-stem sorghum SKS and a juicy-stem sorghum MS3B, Fujimoto et al. (2018) finely mapped the *D* locus to an 18.99-kb interval on chromosome 6, and one gene, *Sobic.006G147400* (referred to as the *D* gene), was confirmed as the candidate gene controlling the juicy content in sorghum. The *D* gene is a NAC domain transcription factor that can trigger the programmed cell death (PCD) of stem parenchyma cells, which leads to dry and pithy stems in sorghum. All the investigated wild sorghum and dry-stem sorghum cultivars possess a functional *D* gene, while six nonfunctional alleles of the *D* gene were discovered in juicy-stem sorghum accessions. Haplotype distribution of the *D* gene was found in both African and Asian germplasms. Two nonfunctional alleles were only distributed in African germplasms, which indicated that a nonfunctional *D* gene could have been selected at an early stage in Africa.

In another study, the same underlying gene (referred to as *Dry*) in the *D* locus was also identified using GWAS and map-based cloning (Zhang et al. 2018). The authors discussed more about the relationship between *Dry* selection and the origin of domesticated sweet sorghum. Principal component analysis (PCA) based on the whole-genome resequencing data of 241 sorghum accessions collected worldwide revealed that the wild, pithy, and juicy accessions were clustered into three different groups, although there were some exceptions in both the pithy and juicy groups. *Dry* acts as a master transcription factor that can regulate a series of genes involved in cell wall biosynthesis and loss of function of the *Dry* gene in juicy sorghum causes irregular parenchyma cells and thinner secondary cell walls. A positive selection signal was detected in the juicy sorghum subgroup, which had a significantly lower π value than the pithy subgroup. Twenty-three haplotypes of the *Dry* gene were found in 42 wild sorghum lines with pithy stems. Two of them were discovered in 86 landraces exhibiting dry pithy stems, and 112 improved cultivars carried four nonfunctional haplotypes. This variation in *Dry* gene haplotype diversity indicates that a bottleneck effect exists in the *Dry* gene during sweet sorghum domestication. In addition, the *Dry* locus also exists in collinear genomic regions in other cereal crops, such as rice, wheat, millet, and maize, indicating that the *Dry* locus is an important target in designing crops with both high grain yield and high stem biomass.

### Flowering

Sorghum is a short-day C4 grass with photoperiod-sensitive characteristics that evolved in a tropical, equatorial region. It adapted to long-day environments after dispersing to high latitudes and temperate regions. Early flowering was generally selected for grain sorghum to ensure reproduction by avoiding drought or low temperature, while other sorghum types, such as sweet sorghum, forage sorghum, and energy sorghum, were selected to have a longer duration of vegetative growth to acquire a higher biomass yield. The diversification of sorghum flowering time under long-day conditions indicates that multiple genes could be selected to produce photoperiod-insensitive varieties. Breeders have paid close attention to identifying genes controlling sorghum maturity, and a series of maturity loci (named *Ma1* to *Ma6*) have been reported over the last century (Quinby and Karper 1945; Quinby 1966, 1967; Rooney and Aydin 1999). All six loci are dominant in suppressing flowering under long-day conditions, and the *Ma1*, *Ma2*, *Ma3*, *Ma5*, and *Ma6* loci were identified in these years, which allowed us to obtain a better understanding of their related genetic basis.

Using a BC_1_F_1_ population and another F_2_ population, *Ma1* was fine-mapped to an 86-kb interval on chromosome 6, and *Sb06g014570* (*SbPRR37*) was confirmed as the candidate gene (Murphy et al. 2011). *SbPRR37* encodes a pseudoresponse regulator, and its expression has two peaks in the morning and evening on long days instead of only one morning peak on short days. The higher expression level of *SbPRR37* on long days activates the expression of the floral inhibitor gene *CONSTANS* (*CO*) and represses *SbEhd1* (*Early Heading Date 1*), which ultimately downregulates *FT* genes (*SbCN8* and *SbCN12*) expression. *SbCN8* and *SbCN12* are florigen genes that play substantial roles in inducing flowering time (Turck et al. 2008; Yang et al. 2014b). Three nonfunctional alleles were found in early-flowering accessions cultivated in long-day conditions, which indicates the possible multiple origins of photoperiod-insensitive sorghum in diversification progress when sorghum spreads to temperate regions. Another association analysis study of sorghum maturity also identified the *SbPRR37* gene by using GWAS in a sorghum mini-core collection (Upadhyaya et al. 2013). Multiple variant alleles of the *SbPRR37* genomic sequence from 253 landraces and historic sorghum cultivars were found. Some alleles dominantly distributed in a specific sorghum race or geographic region reveals the selection history and gene flow of *PRR37* when sorghum was introduced to high latitudes in a new continent by human activity. For example, *prr37*^*Kafir−1*^ and *prr37*^*Kafir−2*^ alleles were mainly distributed in the Kafir race, and *prr37*^*Durra*^ was dominant in Chinese kaoliang (Klein et al. 2015).

*SbGhd7*, encoding a protein containing a CCT domain (CONSTANS, CO-like, and TOC1), was identified as the candidate gene in the *Ma6* loci (Murphy et al. 2014). Its orthologous gene in rice, *GHD7*, inhibits the expression of *EHD1*, which regulates the expression of *Hd3a* (*FT* gene in rice) in response to day length. *SbGhd7* has a similar expression pattern to *SbPRR37* and inhibits the expression of *SbEhd.* Two recessive *ghd7* alleles were found in photoperiod-insensitive cultivars. The dominant alleles of *SbGhd7* and *SbPRR37* act in an additive fashion to delay flowering under long-day conditions. Other studies confirmed that *phytochrome B* (*PhyB*) is the causal gene in *Ma3* loci, and *phytochrome C* (*PhyC*) was proposed as the underlying gene for *Ma5* (Childs et al. 1997; Yang et al. 2014a)*.* Both *PhyB* and *PhyC* have been reported to be related to flowering regulation in a light-dependent manner in *Arabidopsis* and rice. Genetic evidence revealed that *PhyB* is epistatic to *Ma1* (*SbPRR37*) and *Ma6* (*SbEhd*). PHYB inhibits the expression of *SbEhd1*, which activates the expression of *SbCN8* and *SbCN12* under long-day conditions. Another sorghum *FT* gene, *SbCN15*, is also repressed by *PhyB* regardless of photoperiod. The underlying gene of *Ma2* was finely mapped as *Sobic.002G302700*, which encodes a lysine methyltransferase with a SET and MYND (SYMD) domain (Casto et al. 2019). It enhances the expression of *SbPRR37* and *SbCO*. A genetic interaction between *Ma2* and *Ma4* was observed. Two recessive *ma2* alleles were discovered in early-flowering sorghum lines under long-day conditions. These studies shed light on the scenario of sorghum diversification and improvement under human selection for adaptation in temperate regions.

### Awn

The awn is a needle-like structure that extends from the lemma and is very common in gramineous crops, such as wheat, barley, rice, oats, and sorghum (Gu et al. 2015). There are some advantages to having awns for wild species. For example, the awn can prevent insects and birds from predating the seeds, while a barbed awn can help the seeds efficiently spread by sticking to animal furs (Hua et al. 2015; Jagathesan et al. 1961). It has also been reported that awns can contribute to yield by producing more photosynthate in wheat and barley (Du et al. 2021). In wild wheat, awns can help the seeds germinate by pushing them into the soil. Awns can bend or twist when the surrounding humidity changes and therefore produce the mechanical force needed to orient the spikelet (Elbaum et al. 2007). However, long cultivar awns cause difficulties in harvest, processing, and storage. As a result, breeding short-awn or awnless varieties occurs during crop domestication and improvement.

In addition to the normal seed-bearing spikelets, sorghum inflorescence has sterile pedicellate spikelets. It has been proven that the sterile spikelets in sorghum, not the awn, can have the capacity for photosynthesis (AuBuchon-Elder et al. 2020). Therefore, sorghum awns are likely not a carbon source, although all wild sorghum species have long awns. Girma et al. (2019) conducted a GWAS of the presence or absence of awns using 1425 Ethiopian landrace accessions. The GWAS results revealed a leading peak at 72.6 Mb on chromosome 3 and identified eight significant SNPs (Girma et al. 2019). However, no genetic confirmation was performed in this study, and the underlying mechanism remains unclear.

A recent study identified a major gene, *Awn1*, which is responsible for awn loss in cultivated sorghum (Zhou et al. 2021). In this study, Zhou et al. finely mapped the *awn1* gene through a RIL population constructed from a cross between the wild sorghum progenitor *Sorghum virgatum* (SV) and the improved sorghum cultivar Tx623. After narrowing down the interval into a 9.5-kb region on chromosome 3, sequence comparison showed a large 5.4-kb insertion in the domesticated sorghum cultivar Tx623. Only one gene, *Sobic.003G42130*, was annotated in this insertion, which was the same candidate gene in the abovementioned GWAS. *Sobic.003G42130* was named *awn1* and was proven to be derived from an ancestral homologous gene on chromosome 10, *Sobic.010G225100*, which was referred to as *awn1-10*. *Awn1* encodes the identical protein with the ALOG domain but recruits a new promoter and has a higher expression level than *awn1-10*. Transcriptional activity assays and yeast two-hybrid assays proved that Awn1 is a transcriptional repressor. RNA-seq and DAP-seq analysis revealed that it can downregulate some MADS-box genes involved in flower development and the orthologous genes of *DL* and *LKS2* of rice, leading to a reduction in awn elongation in sorghum. Genomic sequence comparison found that this 5.4-kb fragment on chromosome 10 between SV and Tx623 had more SNPs than between *awn1* and *awn1-10* in Tx623, indicating that duplication on chromosome 3 could have occurred after domestication. *Tajima’s D* test revealed a significant selection signal in the neighboring regions of *Awn1,* and the awnless sorghums had the lowest genomic diversity around this region. *Awn1* is a largely effective gene for more than 30% of phenotypic explanations of awn presence or absence in a natural sorghum population, which could be used in further awnless sorghum breeding. Homologs of *Awn1* could be further exploited in other cereals, and it is unclear whether it also experienced similar parallel selection.

### Glume coverage

Wild sorghum has a pair of tenacious glumes that cover the seed, which can protect the seed from being infected by fungi, birds, and insects in the natural environment. However, it caused a huge obstacle for threshing during sorghum domestication. In agricultural practice, seeds tightly covered by glumes pose difficulties for modern automated planting, threshing, and processing (Adeyanju et al. 2015). As a result, farmers favor sorghum grains with low glume coverage, which induces enriching variations in glume coverage in modern sorghum accessions. The spikelet morphology of sorghum differs from that of rice and maize. In rice, the glumes degenerated. The revolved hard lemma and palea act as glumes to cover the rice seed. In maize cultivars, the seeds are naked, and the whole ear is covered by bracts (Wu et al. 2019). The different spikelet structures indicate that sorghum could acquire a distinct regulatory network to control glume coverage during domestication.

Recently, Xie et al. (2022) identified a major gene located on chromosome 1, *GC1*, which controls sorghum glume coverage using GWAS and positional cloning. Five main haplotypes were identified from 482 sorghum accessions, named WT *GC1*, and mutated *gc1-a*, *gc1-b*, *gc1-c,* and *gc1-d*. Among them, *gc1-b*, *gc1-c,* and *gc1-d* were rare, while the *GC1* (71%) and *gc1-a* (24%) haplotypes were dominant in the evaluated accessions. The association test demonstrated that *GC1* variation was highly associated with glume coverage instead of yield-related traits (seed length, seed width, and thousand seed weight). *GC1* encodes a protein of 198 amino acids with a Gγ-like domain (referred to as GC1-G) and a predicted transmembrane domain (GC1-T), while *gc1-an* obtains a stop codon at amino acid position 137 but reserves the entire GC1-G and GC1-T domains. The truncated gc1-a exhibited much lower glume coverage than WT GC1. Overexpression of *GC1* reduced glume coverage in independent transgenic lines. Interestingly, knocking out *GC1* increased glume coverage, which was different from what was observed in *gc1-a*, indicating that the truncated protein could still function during the regulation of glume coverage. *gc1-a*-overexpressing plants confirmed this hypothesis with significantly reduced glume coverage compared with *GC1*. These results indicated that both GC1 and the truncated gc1-a negatively control glume coverage in sorghum. Overexpressing or knocking out the orthologous gene of *GC1* in millet also supported this conclusion.

Further study confirmed that the truncated C-terminus of GC1 caused a higher protein accumulation in vivo, which inhibited glume cell proliferation by downregulating cyclin-CDK-related genes. An interacting phospholipase protein, SbpPLAII-1, identified by immunoprecipitation-mass spectrometry (IP-MS), promoted the expression of cyclin-CDK-related genes and longer glumes. SbpPLAII-1 could be degraded when GC1 or gc1-a was accumulated. The more stable gc1-a accelerated this degradation process compared with GC1. These results demonstrated that naturally truncated variations of GC1 contributed to lower glume coverage in sorghum by degrading SbpPLAII-1 and downregulating the expression of cell division-related genes. *Tajima’s D* test showed a significant selection signal in landraces and improved cultivars with low glume coverage. In addition, the nucleotide diversity in the exon 5 and 3’UTR of *GC1* was also significantly reduced in the naked landraces and improved lines compared with wild sorghum. The geographic distributions of five different haplotypes in this study also indicated that the Sahelian zone is a domestication center of sorghum.

### Tannin content

Proanthocyanidins (PAs) are condensed tannins and are products of the flavonoid biosynthesis pathway. They have astringent properties and are present in sorghum grains but absent in other main cereal crops, such as corn, wheat, and rice (Wu et al. 2012). Tannin evaluation of 11,577 cultivated sorghum accessions showed that tannin and non-tannin types were present in approximately equal proportions (45% and 55%, respectively) (Wu et al. 2019). However, it is unclear why domesticated sorghum still preserved these bitter chemicals under human selection. An early study reported that the presence of tannin in sorghum grain was regulated by two genes (*B1* and *B2*) (Smith and Frederiksen 2000). Wu et al. (2012) identified the *Tannin1* (*Tan1*) gene involved in condensed tannins biosynthesis in sorghum. In 2019, two studies identified the same locus related to the presence of tannin in sorghum and shed light on the underlying regulatory mechanism between tannin content and bird feeding behavior.

Xie et al. (2019) first collected phenotypic data by evaluating bird-preference and bird-avoidance characteristics from the field and identified a significant SNP within the *Tan1* gene involved in flavonoid and PA biosynthesis by GWAS. This locus was stably detected regardless of the phenotype of tannin content or by bird damage levels under different populations and field conditions, indicating that there was a relationship between tannin content and the level of bird damage. This was proven because bird-preference sorghum accessions had significantly reduced levels of metabolites involved in anthocyanin and PA biosynthesis. Two mutated alleles, *tan1-a* and *tan1-b*, with no detected tannins were associated with a much more severe bird damage phenotype compared with the wild-type *Tannin1* (*Tan1*). Sparrow feeding experiments demonstrated that sparrows fed on fewer seeds coated with malvidin or PA than untreated seeds. On the other hand, bird-preference seeds with *tan1-a/b* alleles produced more fragrant volatile organic compounds, such as 1-octen-3-ol and hexanal, which caused a longer residence time for sparrow feeding. It is known that the WD40 protein can form a ternary complex by interacting with MYB and basic helix-loop-helix (bHLH) proteins to control multiple biological processes (Ramsay and Glover 2005). Further studies revealed that *tan1-a/b* likely promoted more fatty acid-derived volatiles than *Tan1*, possibly by repressing the expression of *SbGL2*, a key negative regulator of fatty acid biosynthesis.

Wu et al. (2019) reported that *Tan1* and another gene, *Tannin2* (*Tan2*), were both involved in sorghum tannin content regulation. It was identified by QTL mapping and a combined GWAS. Using the different bird damage levels as phenotype data, five significant loci were identified from QTL mapping of a RIL population derived from a cross between P898012 and Tx430. Three of them were related to plant height, but only two significant loci located on chromosomes 4 and 2 were left when using the tannin presence as the phenotype data. These two loci were also identified in the GWAS using tannin content as the input data. The mapping region on chromosome 4 contained *Tannin1*, which has been proven to regulate tannin presence in sorghum grains in a previous study (Wu et al. 2012), and the allele carrying a 1-base pair insertion (referred to as *tan1-a*) in the coding sequence caused the non-tannin grains in Tx430. After sequence variation analysis, *Sobic.002G076600* was considered the candidate gene of *Tan2*. This was also confirmed by complementing the *Arabidopsis tt8* mutant with modified seed pigmentation (Nesi et al. 2000). A 5-bp insertion in *Tan2* (encoding a protein with bHLH domain) caused a frameshift in Tx430 and was denoted as *tan2-a*.

Tannin and non-tannin plants are segregated at a ratio of 1:3 in the RIL population. These results indicated that *Tan1* and *Tan2* were the underlying genes of the *B1* and *B2* loci, respectively, and sorghum grain tannin was present only when both genes were dominant. The recessive alleles, *tan1-b*, *tan1-c,* and *tan2-b*, *tan2-c,* of each gene were discovered from 88 non-tannin accessions, with *tan1-b* and *tan2-b* at a low frequency. *tan1-a* was mainly distributed in East and West Africa, while *tan2-a* dominated in South and West Africa. In addition, *tan1-c* and *tan2-c* were mainly present in South and East Africa, and no *tan2-a* allele was observed in East Africa. These results indicate that non-tannin sorghum could have multiple domestication origins. Further study confirmed that tannin sorghum had a higher proportion in East and South Africa where bird damage was severe, while non-tannin sorghum dominated in West Africa where bird threats were mild. Interestingly, the geographic distribution of tannin sorghum was also connected to human TAS2R variants. It was assumed that humans carrying a TAS2R haplotype were insensitive to bitter tastes, thus contributing to the selection of tannin sorghum to address local severe bird threats in East and South Africa. In contrast, humans carrying another TAS2R haplotype could perceive a bitter taste and prefer non-tannin sorghum in West Africa. The interactions among plants, humans, and the environment demonstrate the complexity of tannin content domestication in sorghum.

### Seed shattering

Loss of seed shattering is considered a hallmark of crop domestication (Li et al. 2006). Wild ancestors of modern crops disperse their seeds by forming an abscission layer between the seed and pedicel, which causes seed shattering and helps their seeds fall off into the soil in a timely manner and propagate efficiently. However, seed shattering is an unfavorable phenotype for farmers, since it results in great yield losses and causes difficulties in harvesting. As a result, non-shattering variants were selected during domestication among most crops.

*Sh1* is the major gene controlling sorghum seed shattering (Lin et al. 2012). It is cloned using a large F_2_ population containing approximately 15,000 individuals derived from a cross of a wild sorghum *Sorghum virgatum* (SV) and a domesticated cultivar Tx430. A YABBY domain transcription factor was identified as the candidate gene of *Sh1* loci. Sequence variation in *Sh1* revealed four main haplotypes, including the wild haplotype SV-like *Sh1* (*Sh1*^*SV−like*^) and three domesticated haplotypes, SC265-like *Sh1* (*Sh1*^*SC265−like*^), Tx430-like *Sh1* (*Sh1*^*Tx430−like*^), and Tx623-like *Sh1* (*Sh1*^*Tx623−like*^). Compared to *Sh1*^*SV−like*^, *Sh1*^*Tx430−like*^ has four causal changes in the promoter and the second intron, which leads to a lower expression level. Variations in *Sh1*^*Tx623−like*^ and *Sh1*^*SC265−like*^ cause frameshift mutations, resulting in truncated proteins lacking the zinc finger and YABBY domains. The proportion of these three non-shattering haplotypes varies in domesticated sorghum races. For example, in the investigated sorghum accessions, *Sh1*^*Tx430−like*^ is prevalent in the caudatum race, while all durra races, most guinea races, and approximately half of bicolor races carry *Sh1*^*SC265−like*^. The other haplotype, *Sh1*^*Tx623−like*^, is mainly comprised of kafir and bicolor races and is widely distributed in South and East Africa. These results indicate that non-shattering sorghum could have been simultaneously domesticated in different regions from local relative wild ancestors. Another study found that the three wild sorghum accessions SL129, SL12, and SL32, collected from Kenya, Nigeria, and Tanzania, respectively, could be the ancestors of the non-shattering haplotypes *Sh1*^*Tx430−like*^, *Sh1*^*SC265−like*^, and *Sh1*^*Tx623−like*^, respectively (Wu et al. 2022). Interestingly, the syntenic region harboring orthologous genes related to the non-shattering phenotype in rice and maize was also under positive selection, which illustrates that the orthologous genomic region of *Sh1* underwent parallel selection in different cereal lineages.

*SpWRKY*, identified from a wild sorghum species, *Sorghum propinquum*, is also a major gene for sorghum seed shattering (Tang et al. 2013). Compared to the non-shattering allele *SbWRKY*, *SpWRKY* has a longer translated protein since it recruits a new start codon. However, it seems that *SbWRKY* is not related to domestication. It is more likely that *Sorghum propinquum* keeps the seed shattering by obtaining a functional *SpWRKY* from a shared common ancestor with *Sorghum bicolor*. Intriguingly, the expression of *SpWRKY* in non-shattering RTx430 restored the shattering phenotype. Regardless, the relationships and molecular mechanisms between the two genes remain largely unknown and must be further elucidated.

## Conclusions and perspectives

Crop domestication is a protracted process that entails the early selection of wild progenitors, subsequent diversification when the landrace spreads to a new environment, and modern improvement breeding. In this review, we discussed the origin and classification of five basic cultivated breeds of sorghum. Genomic information reveals that there is no obvious domestication bottleneck in sorghum, which differs from other main crops. We summarized key genes that have recently been reported to be involved in sorghum domestication and elucidated their genetic molecular mechanisms. The underlying genes of these agronomic traits were discussed in detail, and the genomic footprints of these genes give us a better understanding of how plants can achieve ideal traits between the environment and human activity during their evolutionary process.

Cereal domestication started in the Neolithic Age approximately 10,000 years ago. During the evolutionary process of plants, multiple agronomic traits were altered that benefited humans during parallel selection across major crops, which is known as domestication syndrome (Gepts 2014; Stetter et al. 2017). The genes that occur in parallel selection always occupy few but important signaling pathway positions. Loss or gain of function in these paralleled selected genes are conserved in related crop species despite different mutation forms (Lenser and Theissen 2013). Recently, an increase in our knowledge about key genes underlying domestication-related traits has been identified in rice, maize, and wheat (Fernie and Yan 2019), which could inspire research of sorghum domestication. For example, the only identified major gene, *SbTB1*, controlling tiller number in sorghum, is the ortholog of well-known *TB1* in maize (Doebley et al. 1997). However, this does not occur in some homologs of those reported distinguished genes due to distinct selection pressure. Millet *SiGC1*, the ortholog to sorghum *GC1*, did not exhibit a parallel section in naked grains since it already had thin glumes and easy-threshing grains (Xie et al. 2022). It is noteworthy that these key domesticated genes are also located in simple regulatory pathways and have minimal pleiotropic effects, which can prevent additional side effects on other important agronomic traits, such as the flowering time pathway (Turck et al. 2008).

One cost of crop domestication is mutation load, which refers to the increase in deleterious mutations in the genome (Gaut et al. 2018). Domestication also caused a loss of rare or elite alleles that could resist biotic or abiotic stress. Interestingly, domesticated crops or animals can reacquire wild-like traits, which is called the de-domestication process (Wu et al. 2021). In sorghum, the effects of mutation load can be mitigated by occasional hybridization between different sorghum accessions (Brown 2019; Smith et al. 2019). A modern understanding of the molecular mechanisms underlying domestication-related genes and the site-directed mutations by highly-efficient genome editing techniques is ushering in the era of Breeding 4.0 (Wallace et al. 2018). Using advanced biotechnologies such as genome editing techniques, researchers can now rapidly achieve de novo domestication of a crop from its wild ancestors. This could preserve the elite genes lost during human selection (Huang et al. 2022; Lyzenga et al. 2021). Sorghum has the potential to be widely utilized not only for food and forage, but also for industrial raw materials, such as sugar and biofuel. The results of genetic studies and molecular biology provide a better understanding of sorghum domestication at the molecular level. Additionally, the beneficial alleles of the major domestication-related traits could contribute to the efficient and accurate breeding of sorghum.

## Data Availability

Data sharing is not applicable to this article, as no datasets were generated or analyzed during the current study.
